# Ultralight super-hydrophobic carbon aerogels based on cellulose nanofibers/poly(vinyl alcohol)/graphene oxide (CNFs/PVA/GO) for highly effective oil–water separation

**DOI:** 10.3762/bjnano.9.49

**Published:** 2018-02-12

**Authors:** Zhaoyang Xu, Huan Zhou, Sicong Tan, Xiangdong Jiang, Weibing Wu, Jiangtao Shi, Peng Chen

**Affiliations:** 1 College of Materials Science and Engineering, Nanjing Forestry University, Nanjing 210037, China; 2 Jiangsu Provincial Key Lab of Pulp & Paper Science & Technology, Nanjing Forestry University, Nanjing 210037, China

**Keywords:** 3D network structure, carbon aerogel, cellulose nanofibers, graphene oxide, oil absorption, poly(vinyl alcohol)

## Abstract

With the worsening of the oil-product pollution problem, oil–water separation has attracted increased attention in recent years. In this study, a porous three-dimensional (3D) carbon aerogel based on cellulose nanofibers (CNFs), poly(vinyl alcohol) (PVA) and graphene oxide (GO) was synthesized by a facile and green approach. The resulting CNF/PVA/GO aerogels were synthesized through an environmentally friendly freeze-drying process and then carbonized to yield CNF/PVA/GO carbon aerogels with low density (18.41 mg cm^−3^), high porosity (98.98%), a water contact angle of 156° (super-hydrophobic) and high oil absorption capacity (97 times its own weight). The carbonization treatment of the CNF/PVA/GO aerogel not only improved the hydrophobic properties but also enhanced the adsorption capacity and specific surface area. Given the many good performance characteristics and the facile preparation process of carbon aerogels, these materials are viable candidates for use in oil–water separation and environmental protection.

## Introduction

In recent years, oil spills and oily industrial wastewater have attracted worldwide attention and have threatened the survival of human beings. Therefore, the development of processes and materials that can effectively separate oil and water is urgently needed. Among the existing techniques used for oil recovery, absorption is considered to be an economical choice because of its physical collection, lower costs, simplicity and high efficiency [1–2]. However, traditional sorbents, such as various synthetic organic polymers, natural organic materials, inorganic sorbents and polymer-based composites, tend to suffer from low adsorption capacity, secondary pollution and poor selectivity [3–4]. Therefore, there is a demand for the synthesis of renewable, effective and environmentally friendly sorbent materials to address the oil spill accidents.

Cellulose is considered to be the most abundant renewable natural polymer on the planet because of its biodegradability, sustainability, nontoxic nature, and biocompatibility [5]. Cellulose nanofibers (CNFs) derived from cellulose have gained wide attention due to their outstanding mechanical properties [6–7] such as an elastic modulus of 140 GPa [8]. In an aqueous environment, CNFs with a high aspect ratio and high surface area have potential for forming a 3D network structure. As a result, research on cellulose aerogels has attracted more and more attention because of its outstanding properties such as high porosity, low thermal conductivity and low density [9–10]. Therefore, CNFs can potentially be an excellent nanofiber for high-performance polymer nanocomposites, thereby presenting unprecedented opportunities for the development of durable engineering materials from a renewable resource for structural, consumer, and medical applications [11–12]. There is a long history of using plant cellulose fibers as reinforcements in polymer composite materials [13–14]. However, the use of nanoscale cellulose fibers to reinforce polymers is a relatively recent effort [15–16]. Despite the challenges described below, CNFs have been combined with various polymer matrices including thermoset resins [17–18], elastomers [19–20], polymer latexes [21], water-soluble/dispersible polymers [22–23] and biodegradable polymers [24].

Although CNF aerogels have many excellent characteristics, their strength and modulus are lower than other inorganic polymer materials. As a consequence, we report a CNF-based aerogel (CNFs/PVA/GO aerogel) composed of a CNF skeleton combined with PVA and GO, which could conquer these drawbacks. PVA is a polymer that has many excellent properties such as biodegradability, low cost, biocompatibility and water solubility [25]. Additionally, GO was chosen as the second component in the CNF-based aerogel due to its excellent mechanical properties. Besides, the oxygen atoms on its surface can promote the formation of hydrogen bonds among the three aerogel components (CNFs, PVA, and GO). The CNF/PVA/GO aerogels show outstanding thermal and mechanical properties but also exhibit some drawbacks such as poor hydrophobic properties and low oil absorption capacity. Therefore, a carbonization treatment of the CNF/PVA/GO aerogels was required to improve their properties.

In this work, we present a facile process for preparing carbon aerogels exhibiting super-hydrophobicity for oil–water separation. The CNF/PVA/GO aerogel with a macroscopic 3D structure was prepared through freeze drying to connect the three components. Then, the super-hydrophobic CNF/PVA/GO carbon aerogel was fabricated by a carbonization treatment in a tubular furnace. The as-prepared carbon aerogel exhibited a porous structure and super-hydrophobicity and showed high selectivity and outstanding oil absorption capacities for various types of organic solvents and oils. Considering its inexpensive raw materials, green synthetic method, and high performance, the CNF/PVA /GO carbon aerogel shows great potential in the treatment of oily wastewater and oil spill cleanups.

## Results and Discussion

### Morphology and microstructure of carbon aerogels

**Figure 1:**
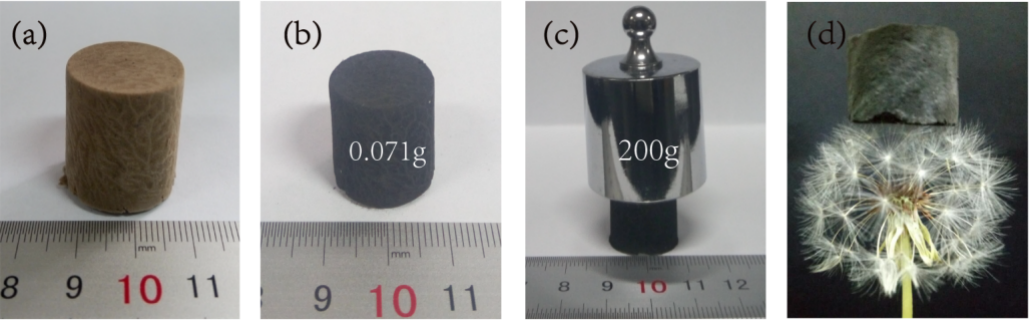
Digital image of (a) CNF/PVA/GO aerogel with dimensions of 1.6 × 1.7 cm; (b) CNF/PVA/GO carbon aerogel with dimensions of 1.5 × 1.6 cm; (c) the carbon aerogel supporting a mass loading of 200 g; (d) the ultralight CNF/PVA/GO carbon aerogel, supported by a dandelion.

Figure 1a and 1b show digital photographs of the CNF/PVA/GO aerogel and the CNF/PVA/GO carbon aerogel. After the carbonization treatment, the color of the CNF/PVA/GO aerogel was changed from brown to black. In addition, the dimensional contraction rate of the CNF/PVA/GO carbon aerogel was relatively small, which illustrated that the structure was stable. As shown in Figure 1c, the carbon aerogel, which has a weight of 0.071 g, can support 200 g of weight (approximately 2817 times its weight) without any deformation, which is attributed to its interconnected 3D microstructure. The density of the carbon aerogel was approximately 18.41 mg cm^−3^, and the whole aerogel could be placed on the top of a dandelion without causing significant deformation (Figure 1d), indicating its low density.

**Figure 2:**
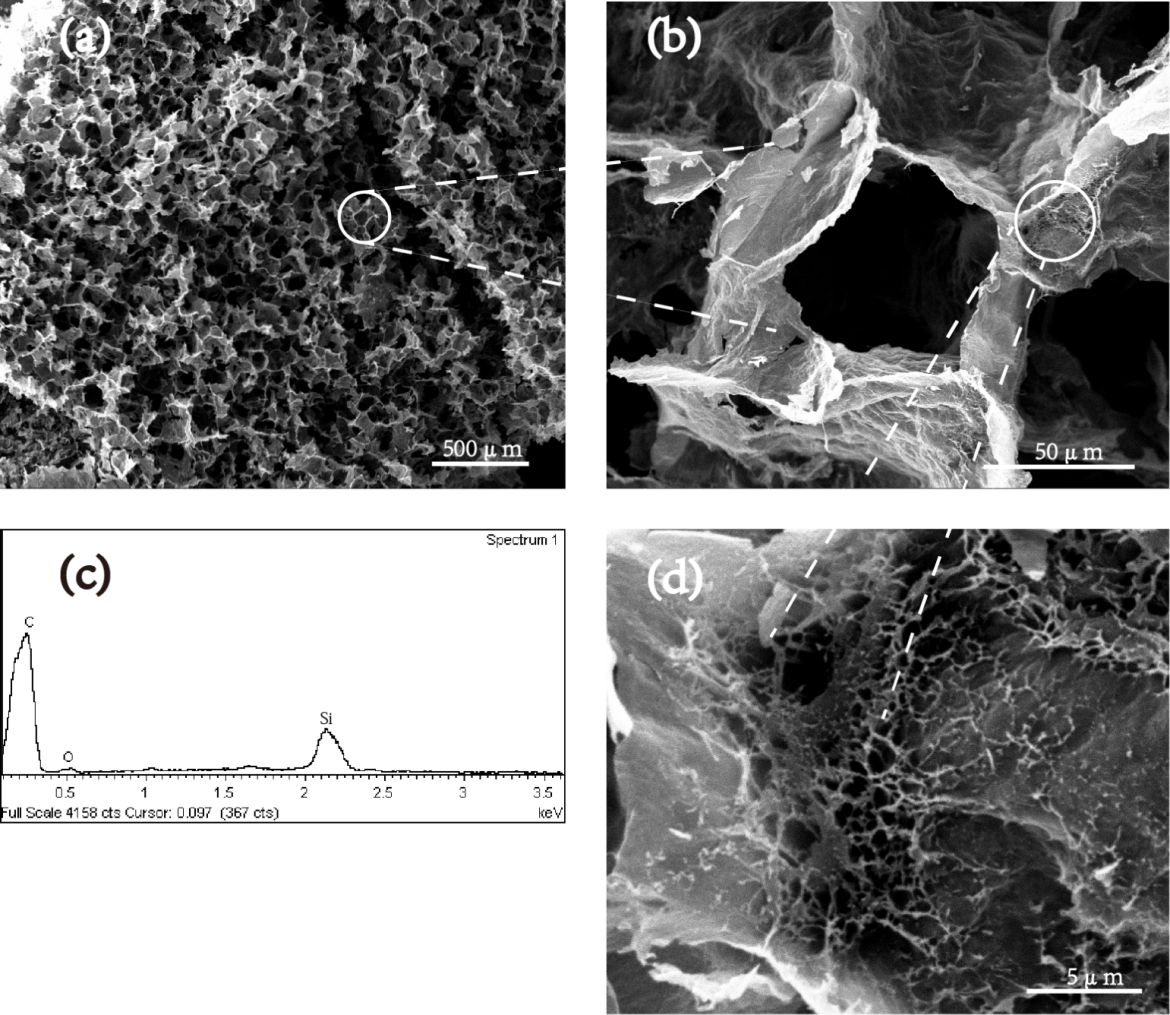
(a), (b), and (d) are SEM images of the inner part of the super-hydrophobic CNF/PVA/GO carbon aerogel; (c) EDS element mapping of the CNF/PVA/GO carbon aerogel.

The microstructure of the CNF/PVA/GO carbon aerogel was observed by SEM (Figure 2a, 2b, and 2d). As shown in Figure 2a, the CNF/PVA/GO carbon aerogel showed porous and interconnected 3D structures with pore size ranging from 50–75 μm. The carbon skeleton originated from GO, and PVA and CNFs was connected to each other to form a porous hole structure. As shown in the magnified view presented in Figure 2d, the carbon skeleton was woven by numerous CNF-derived crossed carbon nanofibers, so as to greatly improve its strength. The porous structure of the carbon aerogel provides sufficient space for the storage of absorbed organic solvents or oils, so that its absorption ability is greatly enhanced. In addition, the crossed CNF-derived nanofibers between the carbon skeleton can provide small pathways for liquid flow as well as enhance the capillary effect of the carbon aerogels, thus contributing to the high adsorption capacity [26]. As shown in the EDS element mapping (Figure 2c), the atomic content of C and O is 88.41% and 11.59%, respectively (the appearance of Si is due to the glass specimen stage). The presence of the carbon element signal demonstrated that the carbonization treatment was complete, which is in good agreement with the FTIR and Raman data.

### Chemical properties

**Figure 3:**
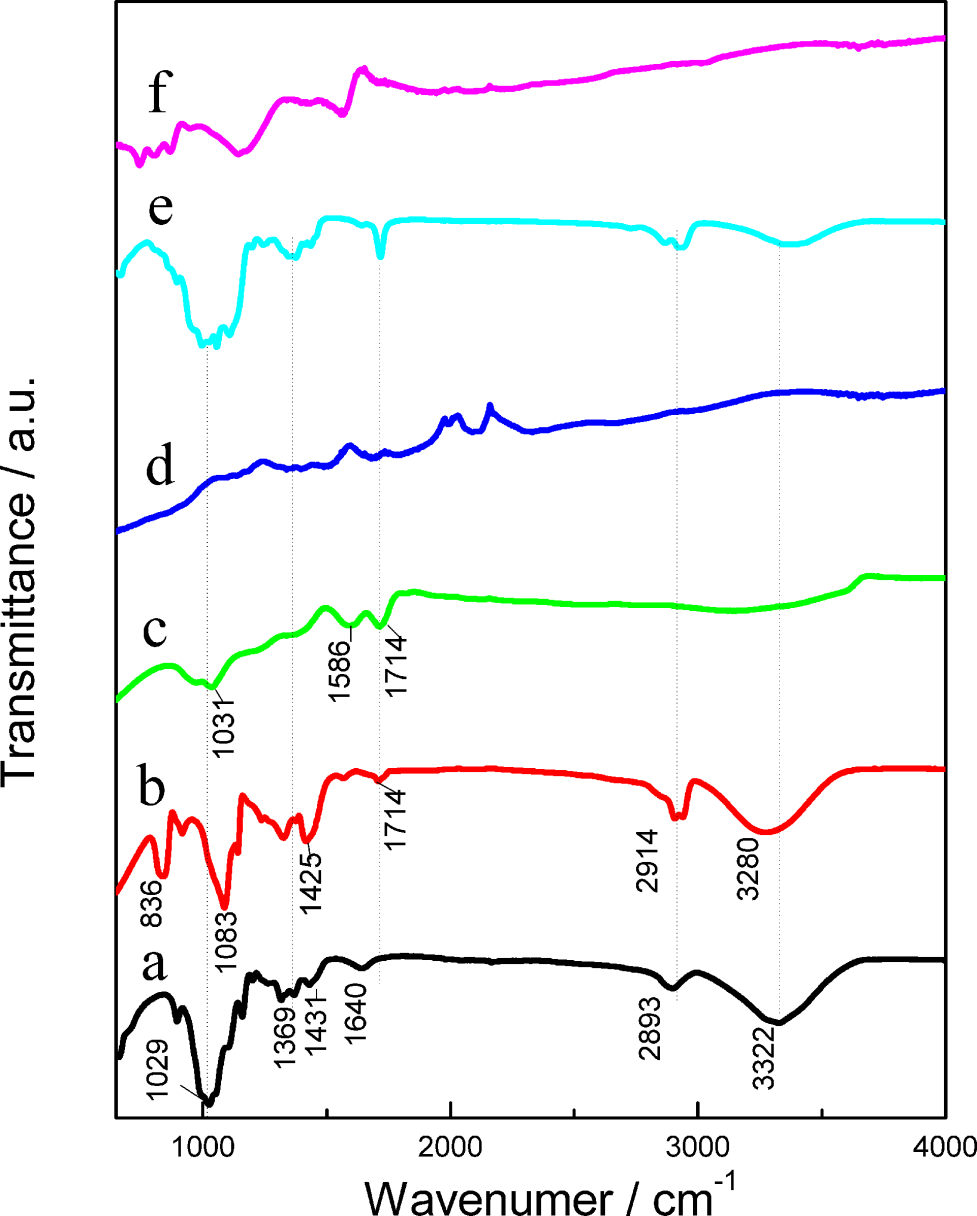
FTIR spectra of the (a) CNFs, (b) PVA, (c) GO, (d) rGO, (e) CNF/PVA/GO aerogel, and (f) CNF/PVA/GO carbon aerogel.

The FTIR spectrum of the CNFs, PVA, GO, rGO, CNF/PVA/GO and CNF/PVA/GO carbon aerogels are shown in Figure 3. The CNFs exhibit FTIR absorption bands around 3323 cm^−1^ (the stretching of hydroxy groups), 2893 cm^−1^ (C–H stretching), 1640 cm^−1^ (H–O–H bending of the absorbed water), 1431 cm^−1^ (–CH_2_ bending), 1369 cm^−1^ (O–H bending) and 1029 cm^−1^ (C–O–C stretching vibrations) [27]. In Figure 3b, pure PVA showed peaks at 3280 cm^−1^ (O–H stretching vibration of hydroxy group), 2914 cm^−1^ (saturated CH_2_/CH_3_ groups, stretching vibration), 1714 cm^−1^ (C=O stretching), 1425 cm^−1^ (CH stretching) and 1083 cm^−1^ (C–O–C stretching vibrations) [28]. In Figure 3c, the characteristic peaks of GO, which are located at 1714 cm^−1^, 1586 cm^−1^ and 1031 cm^−1^, are due to C=O in carboxylic acid and carbonyl moieties, C–OH and C–O vibrations, respectively. As shown in the spectrum of rGO (Figure 3d), most peaks corresponding to the carbon–oxygen functional groups disappeared, which indicates the complete reduction of GO. Figure 3e shows the FTIR spectrum of the CNF/PVA/GO aerogel. Simultaneously, the intensities of all the diffraction peaks decreased remarkably, which may indicate that a strong interaction is present among the three components. After the carbonization treatment, only carbon functional groups were retained in the CNF/PVA/GO carbon aerogel (Figure 3f).This theory has been mentioned in previous studies [19,29], which is in agreement with the SEM results.

**Figure 4:**
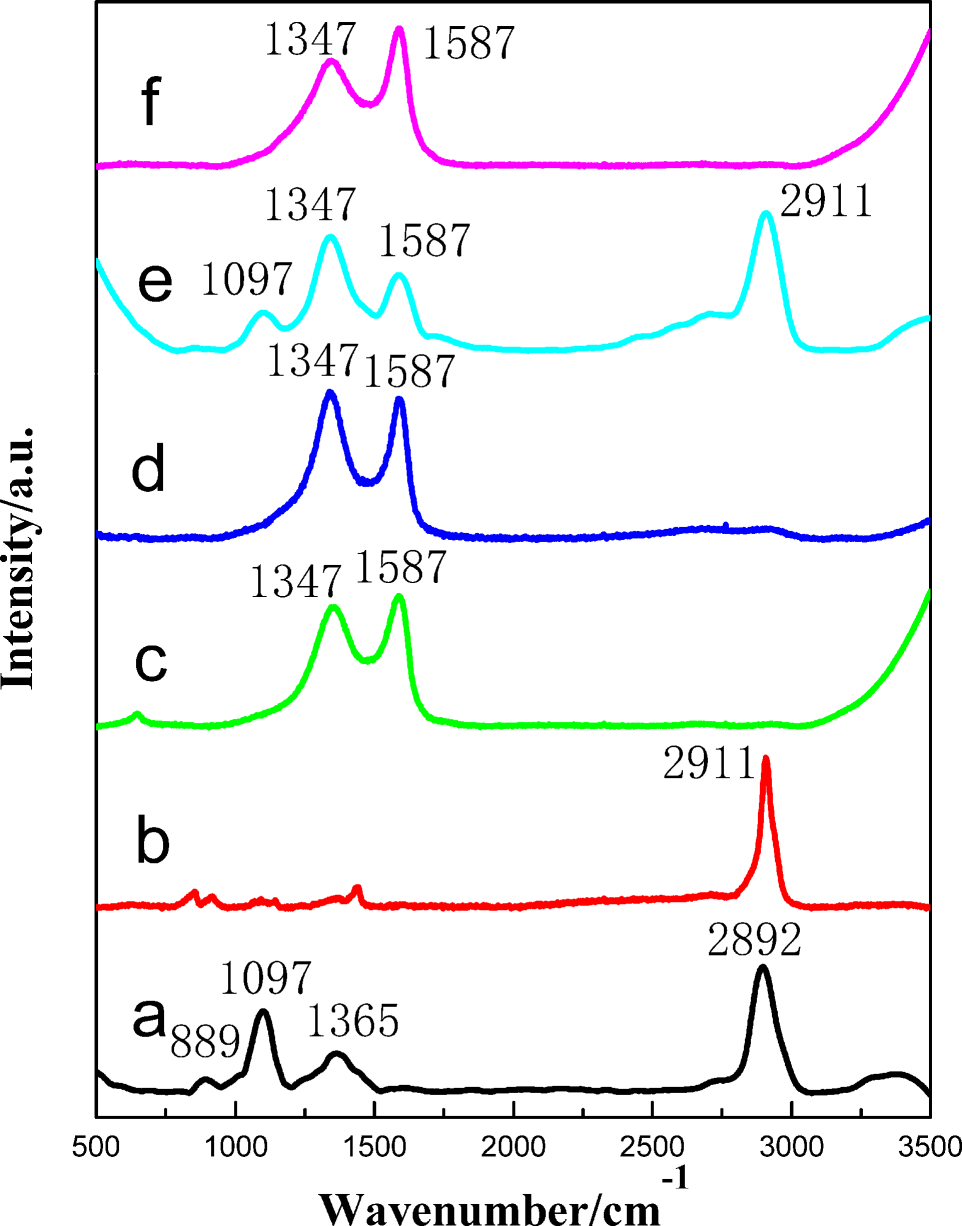
Raman images of the (a) CNFs, (b) PVA, (c) GO, (d) rGO, and (e) CNF/PVA/GO aerogels and the (f) CNF/PVA/GO carbon aerogel.

FTIR can only be used to detect signals from molecules with infrared activity. Therefore, Raman spectroscopy analysis was studied to detect the stretching vibration characteristic peak of homonuclear diatomic pairs. The Raman spectrum of the CNF aerogel is shown in Figure 4a. The bands at 2892 cm^−1^, 1365 cm^−1^, 1097 cm^−1^ and 889 cm^−1^, were due to the asymmetric and symmetric CH_2_ stretching vibrations, the CH_2_ deformation vibrations, the C–O–C glycosidic link asymmetric stretching modes and the in-plane symmetric stretching of C–O–C. In previous work, a similar Raman spectrum was observed for cotton cellulose [30]. Figure 4b shows the Raman spectrum of PVA films. The strong band at 2911 cm^−1^ is due to symmetric and asymmetric CH_2_ stretching vibrations. The Raman spectra of GO and rGO are presented in Figure 4c and Figure 4d, respectively, showing the tangential G-band at 1587 cm^−1^ and the disorder-induced D-band at 1347 cm^−1^ [31]. As is well-known, the G-band corresponds to the tangential vibration of the carbon atoms, while the D-band corresponds to unordered carbon or defective graphitic structures in a Raman spectrum for carbon materials. Besides, the relative intensities of the D-band and G-band (*I*_D_/*I*_G_) of GO and rGO are 0.91 and 1.09, respectively. After the GO was reduced to rGO, a large number of sp^3^ hybrid carbon atoms deoxidized to form a new sp^2^ hybridization area. However, the sp^2^ area of rGO is smaller than that of GO, so the average sp^2^ area of rGO decreases and the ratios increase, as reflected in the gradual *I*_D_/*I*_G_ enhancement observed in the Raman spectra; this result has also been reported in the literature [32–33]. For the CNF/PVA/GO aerogel (Figure 4e), the bands of CNFs, PVA and GO are observed in the Raman spectrum. After the carbonization treatment (Figure 4f), all characteristic bands of the carbon aerogel are tangential G-bands and the disorder-induced D-band, which are in agreement with the SEM and FTIR results.

**Figure 5:**
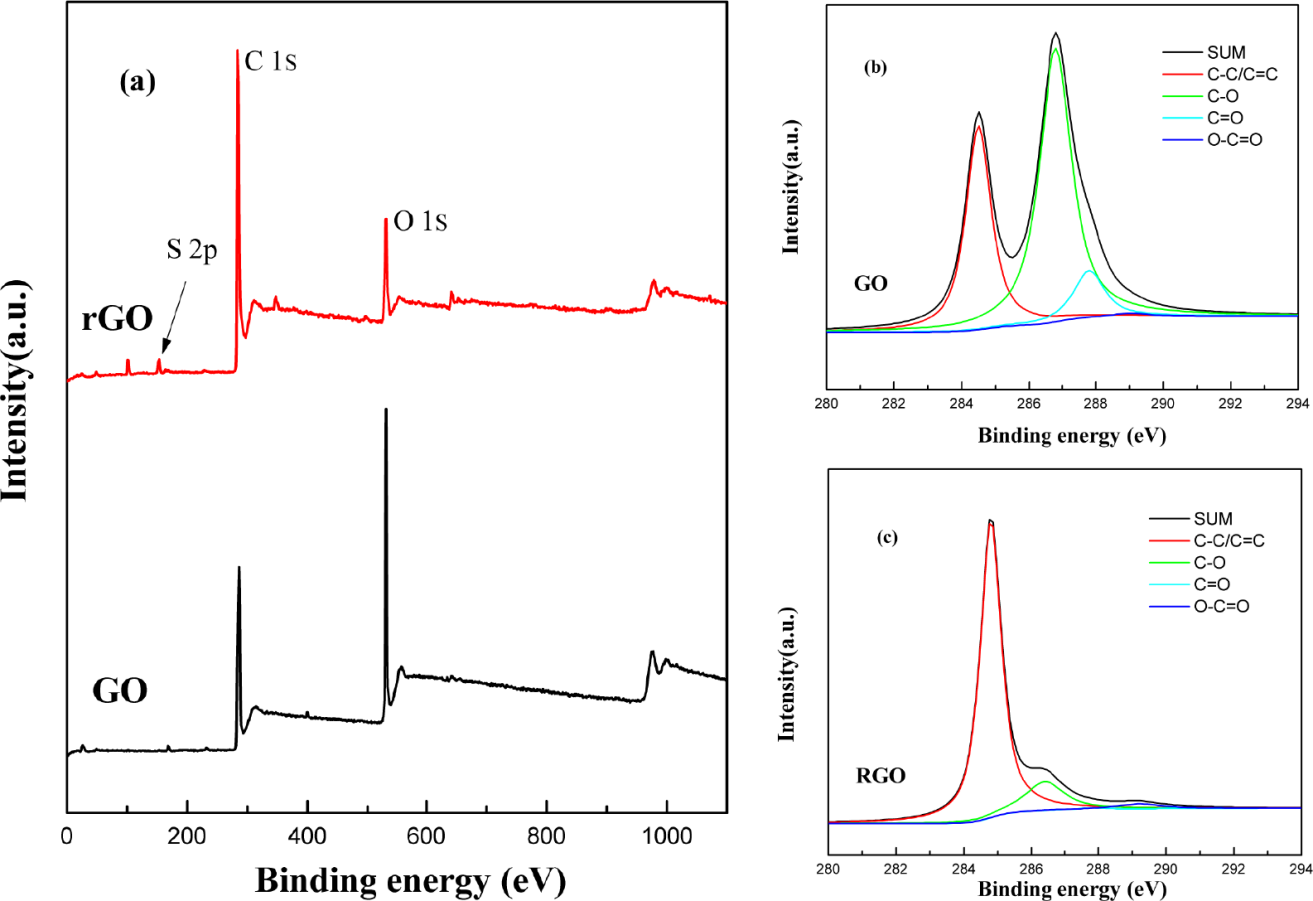
X-ray photoelectron spectrometry spectra and deconvolution of (a) survey scans, and C 1s high-resolution spectra of (b) GO and (c) rGO.

To further confirm the reduction degree of the GO during the carbonization treatment, X-ray photoelectron spectroscopy was carried out (Figure 5a). The higher C/O ratio of rGO revealed the effective reduction during the thiolation reaction, which was verified more clearly in the high-resolution C 1s core-level spectra shown in Figure 5b and 5c. The typical C 1s core-level spectrum was divided into several peaks including C=C (≈284.5 eV), C–C (≈286.8 eV), C=O (≈287.8 eV), and O–C=O (≈289.1 eV) [34–35]. Compared with GO, the peak intensities of C=O, C–O and O–C=O peaks of rGO showed an obvious decrease caused by the ring-opening of epoxide groups and nucleophilic substitution on the hydroxy groups. After the carbonization treatment, the oxygen-containing functional groups in GO were deoxygenated along with the restoration of conjugated structures. Besides, the hydroxy groups on the GO were expected to be significantly eliminated during the thermal decomposition, as was confirmed by the FTIR spectra (Figure 3). It has been reported that the binding energy of hydroxy groups to the carbon atoms is lower than the other oxygen-functional groups, so the hydroxy groups are significantly removed at the initial stage of thermal decomposition [36–37]. The above results and analysis indicated that rGO was successfully prepared, accompanied by a drastic decrease in the number of polar groups and a certain degree of reduction [38].

**Figure 6:**
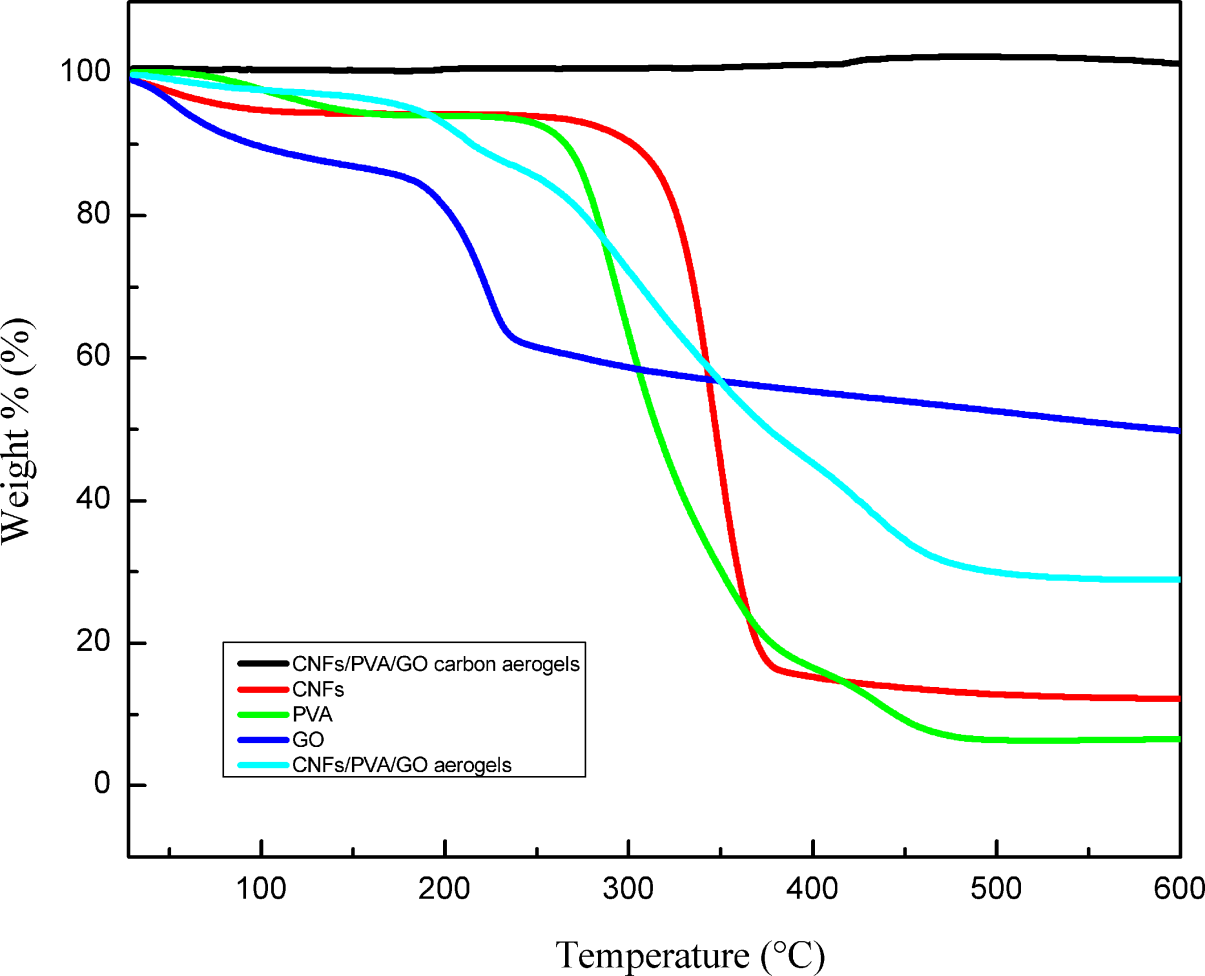
Thermogravimetric analysis (TGA) curves of the CNFs, pure PVA, GO, CNF/PVA/GO aerogel and the CNF/PVA/GO carbon aerogel.

As shown in Figure 6, the thermal stability of the CNF/PVA/GO aerogels before and after the carbon treatment, pure CNFs, pure PVA and GO were measured by thermogravimetric analysis (TGA) in nitrogen from 30 to 600 °C. In addition to the CNF/PVA/GO carbon aerogels, the other three samples showed three different stages of weight loss: the water decomposition stage, polymer decomposition stage, and residual substance decomposition stage. The thermal stability of the aerogel was slightly improved after the introduction of GO. The maximum decomposition temperature of the CNFs after GO introduction increased from 237 to 465 °C, showing that the addition of GO improves the thermal stability of the composite film to some extent. This may have occurred because the GO retarded the thermal decomposition of the aerogels. As shown in the TGA curves of the CNF/PVA/GO carbon aerogels, no weight loss occurred, indicating that carbonation was complete.

**Figure 7:**
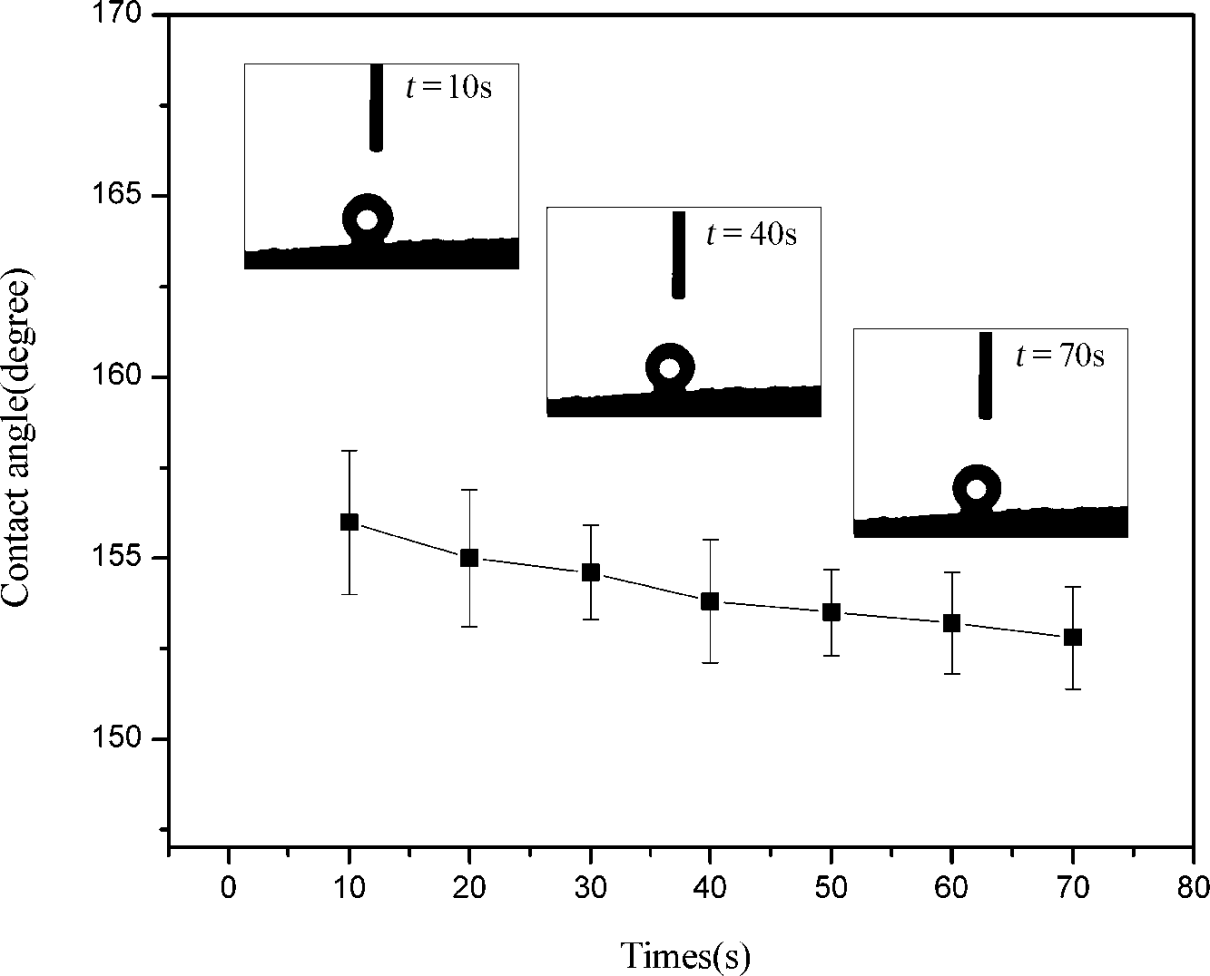
Contact angle of CNF/PVA/GO carbon aerogels.

**Figure 8:**
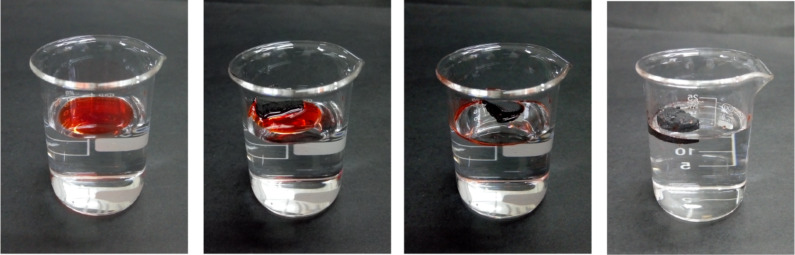
Removal of soybean oil (dyed with Sudan red) from the water surface using carbon aerogel.

Hydrophobicity is an important parameter that influences the absorption capability for organic solvents and oils. Besides, it is reported that the water contact angles are affected significantly by the hydrophilic functional groups on the aerogel surfaces. After the carbonization treatment, the hydrophilic functional groups on the carbon aerogel surfaces disappeared. As shown in Figure 7, the water contact angle of the carbon aerogel was found to be approximately 156°. It is found that the water contact angle on the external surfaces do not show any obvious change with time. These results further confirm the excellent super-hydrophobicity stability of the carbon aerogels examined in this work. Besides, the porous skeleton of the CNF/PVA/GO carbon aerogels provide a large volume for the storage of absorbed organic solvents/oils. As shown in Figure 8, the carbon aerogels can absorb oils (dyed with Sudan red) rapidly and then float on the surface of the water due to its low density, superoleophilic and super-hydrophobic behavior.

**Figure 9:**
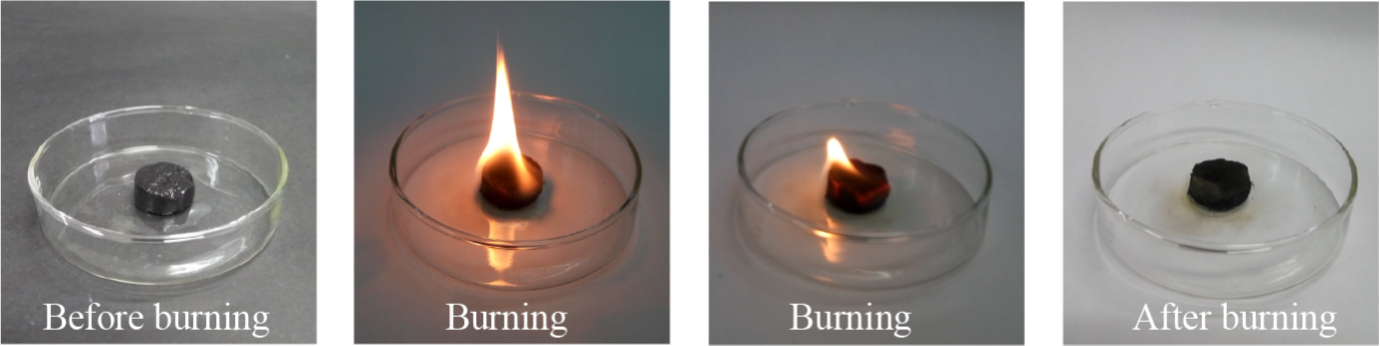
Photographs showing the recycling process of CNF/PVA/GO carbon aerogels via combustion.

**Figure 10:**
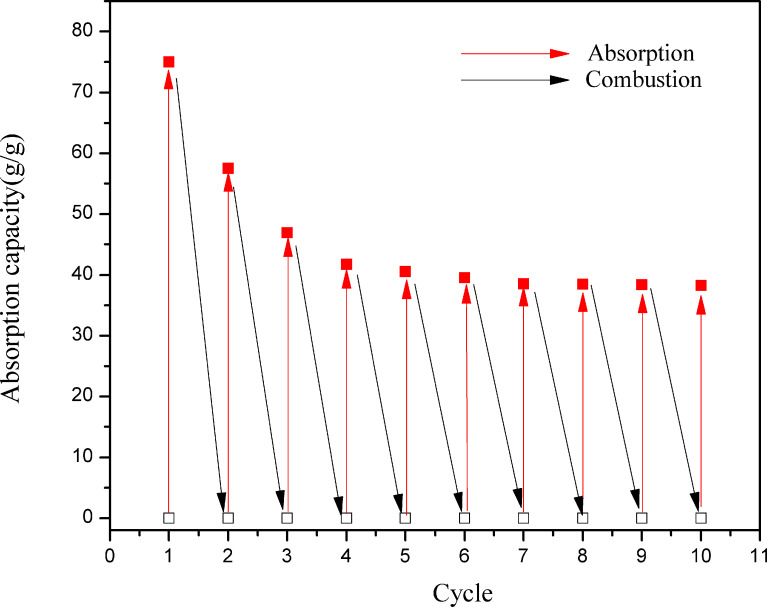
Recycling performance of CNF/PVA/GO carbon aerogels for absorption of soybean oil by absorption/combustion processes.

In addition to high oil absorption capacity, CNF/PVA/GO carbon aerogels must be easily recyclable and regenerable to be put into practical applications. Figure 9 shows the recycling process of CNF/PVA/GO carbon aerogels for absorption of soybean oil by absorption/combustion processes. During the burning process of the carbon aerogels, the basic skeleton is retained, and most of the carbon aerogel did not burn at all, demonstrating their good flame retardance. As shown in Figure 10, the absorption capacity decreases from 75 to 38.2 g/g after 10 cycles, indicating the relatively stable recycling performance of the CNF/PVA/GO carbon aerogel. Furthermore, the porous 3D structure of the carbon aerogel remained unchanged after ten absorption/combustion cycles.

**Figure 11:**
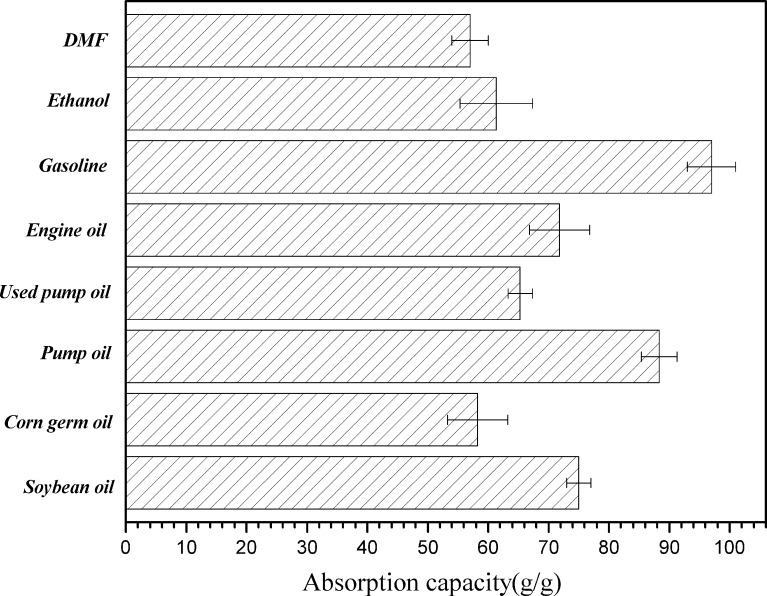
Absorption capacity of CNF/PVA/GO carbon aerogels.

According to the results mentioned above, the absorption parameters were found to play an important role in determining the absorption capabilities of the CNF/PVA/GO carbon aerogels for removal of organic solvents and oils, including *N*,*N*-dimethylformamide (DMF), engine oil, gasoline, ethanol, corn germ oil, pump oil, soybean oil and used pump oil. As shown in Figure 11, the absorption capacity for all the organics and oils were above 57 g/g, especially for gasoline, which attained the highest value at 97 g/g. The absorption capacity and water contact angle of the CNF/PVA/GO carbon aerogel is higher than most existing oil-absorption materials (Table 1). This was attributed mainly to the strong capillary effect of the super-hydrophobic carbon aerogels with high porosity, small hole size and low surface energy. Obviously, the absorption capacity difference of the carbon aerogels was largely determined by the density of the carbon aerogel and surface tension of the solvents and oils. Additionally, the density, which demonstrates the quantity of the holes in the carbon aerogels, plays the more significant role in absorption capacity. The holes in the carbon aerogel provide sufficient space for the storage of absorbed organic solvents/oils, which increase carbon aerogel’s absorption ability.

**Table 1:** Comparison of the absorption capacity and contact angle of different sorbent materials.

Sorbant material	Absorption capacity (g/g)	Contact angle (°)

CNF/PVA/GO carbon aerogels (this work)	57–97	156
silane-treated PVA/CNF aerogel [39]	44–96	150
TMCS/rGO/CNF composite aerogel [40]	33–39	117
spongy graphene [41]	20–86	114
graphene-based aerogel [42]	28–40	–
MTMS-coated cellulose aerogels [43]	18–20	135
super-hydrophobic kapok fiber [44]	54.2–59.8	151
polyurethane/polysiloxane sponge [45]	15–25	157

## Conclusion

To summarize, a super-hydrophobic, lightweight and porous CNF/PVA/GO carbon aerogel has been prepared via a simple freeze-drying process and carbonization treatment. The obtained three-dimensional carbon aerogels exhibited a low density of 18.41 mg cm^−3^ and excellent super-hydrophobicity with a water contact angle of 156°. The super-hydrophobic CNF/PVA/GO carbon aerogels exhibited a high absorption capacity of up to 97 times their own weight for oils and organic solvents. Furthermore, the carbon aerogel also exhibited high absorption selectivity, good recyclability and flame retardance. Our findings are a proof-of-concept for the use of carbonization treatment to provide hydrophobicity for the frame of three-dimension carbon aerogels and offers great potential for applications of these materials in treatment of oil pollution.

## Experimental

### Materials

Bamboo powder was used as the source for the preparation of cellulose and was purchased from Zhejiang Lishui, China. Graphite powder (40 μm), used as the source for GO, was obtained from Qingdao Henglide Graphite Co., Ltd. Poly(vinyl alcohol) (PVA, *M*_w_ ≈ 95,000 g/mol), glutaraldehyde (GA, crosslinker, 25 wt % in H_2_O), potassium hydroxide (KOH), Sudan III, acetic acid (CH_3_COOH), hydrogen peroxide (H_2_O_2_, 30%), potassium permanganate (KMnO_4_), concentrated sulfuric acid (H_2_SO_4_, 98%), hydrochloric acid (HCl), sodium nitrate (NaNO_3_), sodium chlorite (NaClO_2_), phosphorus pentoxide (P_2_O_5_), potassium persulfate (K_2_S_2_O_8_) and ammonium hydroxide (NH_3_·H_2_O) were purchased from Nanjing Chemical Reagent Co., Ltd. These materials were all used without further purification.

### Preparation of carbon nanofibers

The chemical treatment of bamboo powder was prepared by a modified method described in the literature [27]. The detailed experimental steps of preparing the cellulose suspension are documented in our previous paper [40]. Briefly, the suspensions were treated with 2 wt % aqueous KOH to remove pectin, hemicelluloses, and residual starch in the bamboo powder. Then, the samples were treated with a solution of NaClO_2_ and CH_3_COOH to remove lignin. Finally, the samples were treated with a 1 wt % HCl solution to remove the excess metal ions. After the chemical treatment, the slurry of 1 wt % purified cellulose was ground by a grinder to obtain a CNF slurry [46].

### Preparation of graphene oxide

GO was synthesized from graphite powder using a modified Hummers’ method [47–48]. The fabrication process was recorded in detail in our previous research paper [40]. Then, we obtained exfoliated single-layered GO sheets in GO aqueous dispersions under ultrasonication at 80 kHz for 2 h.

### Preparation of poly(vinyl alcohol) solution

PVA (5.0 g) was dissolved in deionized water (100 mL) with continuous stirring for 4 h at 85 °C until the PVA was completely dissolved. Then, the PVA solution was sealed and stored at room temperature for further use.

### Fabrication of CNF/PVA/GO aerogel

**Figure 12:**
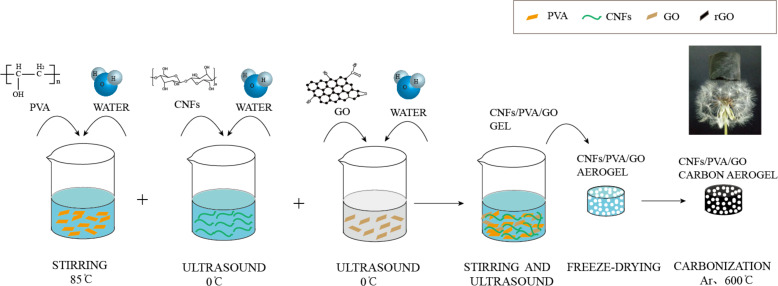
Schematic showing the synthetic steps for preparing super-hydrophobic CNF/PVA/GO carbon aerogel.

The CNF/PVA/GO aerogel preparation process is shown in Figure 12. The CNF solution (8.34 g, 1.2 wt %), PVA solution (3 mL, 0.05 g mL^−1^), and GO solution (4.5 g, 3.34 wt %) were mixed together by vigorous stirring for 1 h. Sulfuric acid (0.8 mL, 1.0 vol %) was then added to the CNF/PVA/GO solution with pH of approximately 5. Then, a glutaraldehyde solution (0.8 mL, 25 wt %) was added to the resulting CNF/PVA/GO solution with mechanical stirring for 1 h, and the mixture was then sonicated in an ultrasonic bath for another hour. Then, the solution was placed in a water bath at 35 °C for 30 min to remove any residual air bubbles. The obtained aqueous gel was subsequently cross-linked/cured in an oven at 75 °C for 3 h. The cross-linked/cured aqueous gel was stored in a refrigerator overnight until completely frozen. Finally, the CNF/PVA/GO aerogels were prepared by a freeze drier.

### Fabrication of super-hydrophobic CNF/PVA/GO carbon aerogel

The carbonization treatment was performed by transferring the obtained CNF/PVA/GO aerogel into a tubular furnace. Firstly, argon gas was introduced for 10 min to remove the air in the CNF/PVA/GO aerogel and tubular furnace. Next, the furnace was heated to 200 °C at a heating rate of 4 °C min^−1^ and sustained for 30 min. Then, it was heated to 600 °C at a rate of 3 °C min^−1^ and held at this temperature for 2 h to assure complete pyrolysis. The following step was to cool it to 200 °C at a rate of 4 °C min^−1^, and finally, natural cooling to room temperature (25 °C) to yield a black and super-hydrophobic CNF/PVA/GO carbon aerogel.

### Sample characterization

A scanning electron microscope (SEM, Quanta 200, FEI, USA) equipped with an energy disperse spectroscopy (EDS) device was used to investigate the surface morphology of the carbon aerogels. The FTIR profiles were obtained using a Fourier transform infrared spectroscopy spectrometer (Nicolet iS10, Thermo Electron Corp., USA). The water contact angle (CA) of the carbon aerogels was obtained using an optical contact angle meter (OCA, Data Physics) with 4 μL water droplets. Raman spectra results were measured using a DXR Raman spectrophotometer (Thermo Scientific, USA). Thermal stability measurements were measured using a thermogravimetric analyzer (TGA, Q 50 TA Instruments, USA). The X-ray photoelectron spectrometry spectra results were carried out using X-ray photoelectron spectroscopy (XPS, AXIS UltraDLD, USA).

The absorption capacity of the CNF/PVA/GO carbon aerogels was measured by immersing the carbon aerogels into various organic reagents and oils for 10 min until saturation. The wet carbon aerogels used for oil absorption were removed from the solution, lifted up for 30 s to allow the surface residual liquid to drip away, and then weighed. The absorption capacity of the carbon aerogels is calculated according to:

[1]
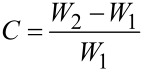


where *C* is the absorption capacity of the carbon aerogels and *W*_1_ and *W*_2_ are the weights of the carbon aerogel before and after absorption. The porosity of aerogel samples was calculated using the following formula:

[2]
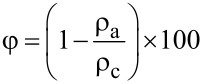


where φ is the porosity of the carbon aerogels, ρ_a_ is the density of the carbon aerogels and ρ_c_ is the density of cellulose (1.5 g cm^−3^) [9].
